# Multifactorial Correlation Analysis of Nursing Unit Staffing Based on Gray Relation Analysis: A Cross‐Sectional Study

**DOI:** 10.1155/jonm/8748353

**Published:** 2026-03-03

**Authors:** Xinyue Pang, Xinmei Cao, Zhi Chen, Jiaqi Shi, Jia Pan, Lijie Mao

**Affiliations:** ^1^ The First School of Medicine, School of Information and Engineering, Wenzhou Medical Medical University, Wenzhou, 325035, Zhejiang, China; ^2^ Respiratory Intensive Care Unit, The First Affiliated Hospital of Wenzhou Medical University, Wenzhou, 325035, Zhejiang, China, wzhospital.cn; ^3^ Cardiac Intensive Care Unit, The First Affiliated Hospital of Wenzhou Medical University, Wenzhou, 325000, Zhejiang, China, wzhospital.cn; ^4^ Operational Performance Department, The First Affiliated Hospital of Wenzhou Medical University, Wenzhou, 325000, Zhejiang, China, wzhospital.cn

**Keywords:** correlation analysis, gray relation analysis, influence factor, nursing staffing, nursing unit

## Abstract

**Background:**

The rationalization of nurse staffing is a complex issue that is influenced by a number of factors. The correlation between many different influences and nursing staffing had not yet been clarified.

**Aim:**

The aim of this study was to use gray relation analysis to analyze the extent to which factors such as human, material, and financial inputs, nursing services, and nursing quality are associated with nursing human resource allocation from a time series and nursing unit perspective, so as to clarify the priorities of nursing unit staffing.

**Methods:**

Based on the previous literature review and expert correspondence, 7 primary and 26 secondary indicators of the factors influencing the staffing of nursing units were identified. Data related to 55 nursing units for the year 2023 were retrospectively collected from the hospital information system and the nursing information system. Gray relation analysis was used to calculate and rank the correlation between each influencing factor and nursing unit staffing in time series and nursing unit series.

**Results:**

Gray relational analysis revealed consistently higher correlation coefficients for primary indicators (time series: 0.72–1.00; nursing unit series: 0.83–1.00) versus secondary indicators (time series: 0.59–1.00; nursing unit series: 0.80–1.00), indicating that the selected manifest variables served as good measures of their underlying latent constructs. Physical/financial inputs and nursing quality/safety‐service outputs ranked highest among primary categories. Key secondary indicators showed strong intercorrelations: actual open beds, nurses on duty, nurse–patient ratio, and nursing work hours for inputs; nursing quality assessment results, bed utilization rate, patient satisfaction, and diagnosis‐related groups for outputs. The correlation patterns for adverse events differed substantially across dimensions, showing higher correlations in unit comparisons than in time series.

**Conclusion:**

This study demonstrates that a multifactor, value‐driven approach using gray relational analysis can effectively identify key inputs and outputs for nursing unit staffing. The findings highlight specific high‐impact indicators for management prioritization, including physical and financial inputs alongside nursing quality and safety outputs. When allocating resources and planning staffing, managers should consider that the strength of relationships for key factors varied significantly between the time‐series and nursing‐unit dimensions. This indicates that the analytical perspective is crucial for identifying critical factors. Future research should focus on developing predictive tools based on these drivers and validating the approach in broader clinical contexts.

**Implications for Nursing Management:**

The correlation analysis of this study provided managers with a reference for evaluating the efficiency of nursing staffing and constructing a nursing staffing model with the priority of different influencing factors.

## 1. Introduction

The shortage of nurses is a global emergency for public health systems. A World Health Organization survey reports that the global shortage of nurses is expected to reach 5.7 million by 2030 [1]. Developing countries in Africa are chronically constrained in terms of nursing human resources due to their level of economic development. Developed countries such as the United Kingdom, the United States, and Japan have a shortage of sustainable nursing human resources. On the one hand, there is a reduction in the age‐appropriate labor force due to accelerated population aging, and on the other hand, the turnover rate of nurses has risen sharply as a result of excessively long working hours and poor remuneration [1]. Based on this, the question of how to achieve efficient staffing of nursing units with limited human resources is an urgent issue. Nursing unit staffing is associated with a variety of factors. Studies have shown that the number or skill mix of nursing staff affects nursing quality, patient prognosis, and adverse patient outcomes [2], while nursing staffing models are constructed based on nurse workload, patient needs, and safe nursing staffing ratios [3]. However, in recent years, relevant studies of nursing staffing have focused more on the economic benefits and safety of several combining factors [4]. Thus, the evidence on whether to increase or decrease the number and mix of nursing staff based on a particular indicator is insufficient. The methods of adjusting nursing unit staffing need to be further explored, as they are influenced by a variety of factors, including nursing unit efficiency, overall economy, and patient disease type. It is unclear how much the different factors in the existing studies have a combined effect on human resource allocation in nursing units. Managers still lack intuitive and rapid multifactorial data analysis to support nursing human resource assessment and adjustment. Gray relation analysis (GRA) is a multifactor statistical analysis method applied to gray relations proposed by Chinese scholar Deng [5]. The method is mainly used to measure the correlation level between discrete sequences and judgment factors [5], which can provide a computational tool for multifactor correlation analysis of nursing unit staffing.

Nursing staffing needs to determine the optimal allocation of nursing resources to meet patient needs and improve the quality of nursing services [6]. It is a complex and comprehensive management problem with many influencing factors, and there are diversity, uncertainty, and ambiguity among the factors. Existing studies have mostly focused on systematic evaluation and qualitative research, while quantitative studies have focused on single‐factor analysis and nursing efficiency evaluation. A systematic evaluation as to the impact of nursing staffing on patient‐, nurse‐, and economic‐related outcome indicators shows that it is not possible to draw conclusions about the effectiveness of the new staffing model on patient, nurse, and cost outcomes due to the low level of evidence [6]. A qualitative descriptive study of semistructured interviews with nurse managers summarizes themes affecting nursing staffing such as government policies, hospital characteristics, patient characteristics, and nurse shortages [7]. Monofactor studies have analyzed the relationship between nurse staffing and factors such as patient prognosis, adverse patient outcomes, and nursing quality. Most of the results show that improving nurse staffing or skill mix can improve patient prognosis and nursing quality [8–10]. Nursing efficiency evaluation mainly takes the number of nurses, the number of beds, and nursing time as input indicators, and the number of discharged patients, the volume of surgery, and the case‐mix index as output indicators, to explore the reasonableness of nursing staffing from the perspective of utilization efficiency or economic efficiency [11]. The above studies have explored the influencing factors of nursing staffing or staffing methods from different perspectives but have not comprehensively considered the relevance of different influencing factors based on the operating conditions of different nursing units. There is still a lack of objective data support for the selection of indicators for staffing methods or model construction.

GRA is widely used in engineering, economics, simulation model validation, and other fields because of its simple calculation method and no requirement of large sample capacity [12]. In medical research, some researchers have gradually applied it to the analysis of disease risk, medical cost, and hospital management factors to provide decision support for doctors or hospital administrators based on the related strength of different factors. Huang et al. [13] use GRA to explore the correlation between the risk of developing dementia and health‐related quality of life influences in diabetic patients, and the results show that the prevalence of sleep disorders in diabetic patients correlated with the degree of depressive symptoms. Peng et al. [14] use GRA and the degree of structural change to explore the changes in the structure of hospital costs for thyroid cancer patients and the reasons for the changes. The results show that the cost of medicines and consumables have a greater correlation with the average hospital cost of patients and suggest that hospitals should strengthen the control of the medicines and medical consumables. Fang et al. [15] use GRA to explore the correlation between the intensity of antimicrobial drug use and the case‐mix index of different medical team leaders, and the results show that managers should focus on the drug use of medical team leaders with high intensity of antimicrobial drug use and low case‐mix index. From this, GRA can clarify the extent to which each factor affects the outcome by describing and comparing the trend of systematic changes, so as to provide managers with a basis for decision‐making. However, this method is still less applied to nursing management research.

Therefore, the aim of this study is to use GRA to analyze the extent to which factors such as human, material, and financial inputs, nursing services, and nursing quality are associated with nursing human resource allocation, so as to clarify the priorities of nursing unit staffing.

## 2. Method

### 2.1. Study Design and Sample

This is a cross‐sectional study using GRA to explore the correlation of factors influencing the staffing of nursing units. The reporting of this study follows the cross‐sectional STROBE guidelines. The study was conducted in a single tertiary Grade A public hospital in Zhejiang Province, China. Such hospitals are among the largest in the country, often comprising dozens of specialized nursing units under centralized management. All 55 nursing units were part of the same hospital. GRA allows for small sample sizes; therefore, a total of 55 nursing units were included in this study as research subjects, of which 29 were surgical nursing units and 26 were medical nursing units. The data collection period was from January to December 2023. Inclusion criteria are as follows: each nursing unit has homogeneity, i.e., each nursing unit needs to admit patients after doctor’s diagnosis and then give appropriate nursing services and treatment during hospitalization; the opening time is more than 12 months and complies with the management system of human resources, nursing quality, and safety formulated by the nursing department of the hospital. Exclusion criteria are as follows: nursing units with changes in nurses, medical resources, and other inputs within the past year due to hospital development planning; incomplete or missing data on factors affecting nursing staffing.

### 2.2. Identification of Factors Influencing Nursing Unit Staffing

Building on literature review and expert consensus, we identified 7 primary and 26 secondary indicators of nursing unit staffing factors, with formal definitions provided in Table 1. These primary indicators serve as latent variables affecting the staffing of nursing units, grounded in Donabedian’s structure–process–outcome (SPO) framework [32] and operationalized through measurable secondary indicators. Manpower inputs, operationalized through staffing ratios such as nurse–patient ratio and absolute nurse counts, quantify foundational workforce structural adequacy essential for service delivery. These personnel resources require complementary infrastructure captured by physical inputs, measured through actual open beds to represent physical care capacity. Financial inputs, manifested in nursing staff costs, then reflect critical monetary investments sustaining human and physical resources. Collectively, these structural elements enable nursing service inputs, gauged by nursing work hours and time‐based metrics to assess clinical labor deployment intensity as the core process converting resources into care. This process generates nursing services outputs, measured through workload complexity metrics including diagnosis‐related groups and operational efficiency indicators such as bed utilization, translating inputs into tangible care volumes. Crucially, such throughput must align with safety and quality outputs, operationalized via adverse events and patient satisfaction to evaluate care effectiveness and safety outcomes. Finally, scientific research outputs, measured by research achievements, assess knowledge‐generation capacity to ensure the system’s adaptive evolution. Collectively, these indicators comprehensively encompass SPO measures explicitly related to nurse staffing—evaluating nursing systems through operational performance, resource allocation efficiency, quality assurance, and sustainable innovation capacity.

**TABLE 1:** Definition of influence factors.

Primary influence factors	Definition of influence factors	Secondary influence factors	Definition of influence factors	Original study authors
Manpower inputs	Allocation and composition of nursing staff resources.	Number of nurses on duty	Refers to the total count of nurses actually participating in clinical work in each nursing unit per day, excluding nurses on sick leave, maternity leave, or other reasons for nonattendance	Huang et al. 2018 [16]
		Number of day shift nurses	Refers to the number of nurses on duty during the day shift	Chang et al. 2020 [17]
		Number of night shift nurses	Refers to the number of nurses on duty at night	Chang et al. 2020 [17]
		Doctor–nurse ratio	Refers to the ratio of doctors to nurses on duty	Chang et al. 2020 [17]
		Nurse–patient ratio	Refers to the ratio of nurses on duty to all inpatients	Griffiths et al. 2018 [18]
		Bed‐to‐nurse ratio	Refers to the ratio of actual open beds to nurses on duty	Kim et al. 2019 [19]

Physical inputs	Tangible infrastructure supporting care delivery	Actual number of open beds	Refers to the number of actual beds opened for the admission of patients	Feng et al. 2021 [20]

Financial inputs	Monetary investment in nursing personnel	Nursing staff costs	Refers to the average income per nurse on duty	Wen et al. 2019 [21]

Nursing service inputs	Directly invested nursing workload	Nursing work hours	Refers to the total number of working hours for nurses on duty	Chen et al. 2022 [11]
		Nursing work hours at night	Refers to the number of working hours at night for nurses on duty	Riman et al. 2022 [22]
		Nursing hours per patient per day	Refers to the average number of nursing hours per inpatient per day, the actual number of working hours for nurses on duty/the actual number of bed days occupied by inpatients during the same period	Peutere et al. 2023 [23]

Nursing services outputs	Clinical workloads and case complexity	Number of diagnosis‐related groups	Diagnosis‐related group (DRG) is a case‐mix classification scheme, i.e., a system of managing patients in several diagnostic groups based on age, disease diagnosis, comorbidities, treatment modalities, severity of disease, etc. This factor refers to the sum of diagnosis‐related groups for cases admitted to different nursing units	Zaranko et al. 2023 [24]
		Case‐mix index	The case‐mix index is the DRG index, which is an average weighting of all inpatient cases, reflecting the technical difficulty and resource consumption in the diagnosis and treatment of patients	Feng et al. 2021 [20]
		Number of relative weights ≥ 2	The relative weight (RW) index is a weight given to each DRG based on its level of resource consumption, reflecting the level of resource consumption of that DRG relative to other diseases. (RW = 1 represents the average level of difficulty of a case and RW ≥ 2 represents the weighting benchmark for difficult cases)	Twigg et al. 2011 [25]
		Actual number of occupied bed days	Refers to the total number of inpatient bed days for all discharged patients	Wei et al. 2018 [26]
		Average length of stay for discharged patients	Ratio of the total number of bed days actually occupied by patients to discharges for the same period	Zaranko et al. 2023 [24]
		Number of bed turnovers	Refers to the number of patient discharges per bed in a given period, number of discharges/number of actual open beds	Coudounaris et al. 2020 [27]
		Bed utilization rate	Refers to the ratio of beds in use to beds available per day, i.e. the total number of bed‐days actually occupied to all bed‐days actually open	Han et al. 2018 [28]
		Number of discharged patients	Refers to the number of all patients discharged after hospitalization, including medically or non‐medically discharged, transfers to other healthcare facilities, deaths	Chen et al. 2022 [11]
		Discharge rate of critical patients	Refers to the number of critically discharged patients as a proportion of the total number of discharges during the same period	Kim et al. 2019 [19]
		Number of surgeries	Refers to the total number of actual surgical operations or treatments performed on inpatients	Trakakis et al. 2022 [29]
		Surgical outputs	Refers to the sum of the number of each surgical operation or treatment in the nursing unit multiplied by the resource‐based relative value scale	Chen et al. 2022 [11]

Nursing safety and quality outputs	Effectiveness and safety of care delivery	Adverse nursing events	Refers to the number of incidents such as pressure ulcers, falls, medication administration errors, unplanned extubation, and catheter‐related bloodstream infections in hospitals	Griffiths et al. 2018 [18]
		Nursing quality assessment results	Refers to the quality assessment score of each nursing unit, including basic nursing care, nursing operation, first aid management, etc	Alenius et al. 2014 [30]
		Patient satisfaction	Refers to inpatient satisfaction scores	van der Mark et al. 2021 [31]

Scientific research outputs	The capacity to generate clinical nursing experience into knowledge	Scientific research achievements	Refers to the quantity of all peer‐reviewed publications, patent applications, approved research funding projects, etc., obtained by nurses after participating in scientific research	Wen et al. 2019 [21]

### 2.3. Data Collection

Data were retrospectively collected from the hospital information system (HIS) and nursing information system (NIS) for all 55 nursing units throughout 2023. Two trained master’s degree nursing students independently entered the data according to the definitions of each influence factor over a 3‐month period. The data were also reviewed weekly by a nursing management specialist to ensure accuracy.

### 2.4. Ethic Considerations

The Review of Ethics Committee in Research of the First Affiliated Hospital of Wenzhou Medical University (KY2023‐291, December 18, 2023) approved this study. We confirm that written informed consent was obtained from all participants involved in this study.

### 2.5. Data Analysis

According to the principle of GRA operation, we used MATLAB R2022a software for data analysis [33]. We explored the intrinsic links between staffing of nursing units and the influencing factors with time series and nursing unit series, respectively. The time series was calculated as the average of all the data related to the nursing unit with the month as an independent group, and the nursing unit series was calculated as the monthly average of the data related to the nursing unit as a group. The specific calculation process included the following steps.

#### 2.5.1. Determination of reference and Comparison Sequences

The standard number of nursing staff, determined on the basis of the Chinese standard bed‐to‐nurse ratio of 1:0.4, will be used as the reference series.

(1) X0=X01,X02,⋯,X0n.

The 26 secondary influences in Table 1 were used as a comparison series.

(2) Xi=Xi1,Xi2,⋯,Xin, i=12,,⋯,m.

#### 2.5.2. Data Dimensionless

In order to eliminate the effect of raw data magnitude, each column of data was homogenized according to the standardization formula.

(3) fX=XX¯,X¯=∑Xn.

#### 2.5.3. Calculate the Difference Sequences, Maximum Difference, and Minimum Difference

The difference sequence is the absolute value of the difference between the reference sequence and each of the comparison sequences, Δ_0i_(*k*) = |*X*_0_(*k*) − *X*_*i*_(*k*)|(*k* = 1, 2, ⋯, *n*). We calculate the maximum and minimum differences, i.e., Δ_max_ and Δ_min_, from Δ_i_(*k*).

#### 2.5.4. Calculation of Correlation Coefficient

(4) λ0ik=Δmin+ρΔmaxΔ0ik+ρΔmax,

where *ρ* is the resolution factor, which usually takes the value of 0.5.

#### 2.5.5. Calculating Correlation

$\gamma_{0i}=\left(1/N\right)\sum_{k=1}^{N}\lambda_{0i}\left(k\right)\left(N=12,,\cdots ,n\right)$, the reference sequence is sorted according to the magnitude of its correlation with each of the comparison sequences.

## 3. Results

### 3.1. Statistical Description

A total of 65 nursing units met the inclusion criteria, and 10 nursing units were excluded due to the presence of relevant missing data or changes in nursing resource inputs. Ultimately, 55 nursing units were included, including 29 surgical nursing units and 26 medical nursing units. The statistically descriptive results of the secondary influencing factors for each nursing unit from January to December 2023 are shown in Table 2.

**TABLE 2:** Statistical description of influence factors.

Secondary influence factors	Mean	Std. deviation	Median	IQR
Number of nurses on duty	15.59	2.01	15.47	2.09
Number of day shift nurses	8.68	1.20	8.76	1.59
Number of night shift nurses	1.23	0.39	1.02	0.07
Doctor–nurse ratio	0.81	0.29	0.81	0.26
Nurse–patient ratio	5.17	0.66	5.16	0.72
Bed‐to‐nurse ratio	3.10	0.40	3.10	0.43
Actual number of open beds	47.55	3.17	49.00	2.00
Nursing staff costs	473,426.75	71,539.98	471,346.70	79,771.63
Nursing work hours	1896.53	244.95	1881.41	253.64
Nursing work hours at night	208.83	65.70	173.60	11.20
Nursing hours per patient per day	2.95	0.34	2.91	0.51
Number of diagnosis‐related groups	39.24	9.80	38.33	12.25
Case‐mix index	1.26	0.57	1.12	0.50
Number of relative weights ≥ 2	438.40	430.20	290.00	530.00
Actual number of occupied bed days	133,622.00	11,151.14	133,809.00	10,168.03
Average length of stay for discharged patients	6.37	2.42	5.99	3.02
Number of bed turnovers	67.04	25.77	60.67	28.15
Bed utilization rate	1.00	0.01	1.00	0.00
Number of discharged patients	267.16	107.22	248.67	118.83
Discharge rate of critical patients	0.17	0.19	0.12	0.20
Number of surgeries	1561.86	1280.63	1445.58	1285.08
Surgical outputs	3,291,972.05	2,854,916.17	3,032,612.83	4,932,333.62
Adverse nursing events	1.12	0.36	1.08	0.50
Nursing quality assessment results	98.88	0.36	98.92	0.50
Patient satisfaction	0.95	0.01	0.95	0.02
Scientific research achievements	1.76	1.21	1.67	1.50

*Note:* All nursing work hours and costs data were collected for the full year (January 1st to December 31st 2023).

Patient satisfaction scores were obtained using the Hospital Consumer Assessment of Healthcare Providers and Systems (HCAHPS) survey, adapted for China’s national standards. The value of 0.95 for patient satisfaction indicated the percentage of patients who rated “overall satisfaction” as greater than 90.

### 3.2. Results of Gray Relation Analysis in Time Series

In the time series analysis, primary indicators (aggregate measures) showed higher correlation coefficients (0.7163–1.0000) than secondary indicators (directly measured variables; 0.5852–1.0000), consistent with their derivation as averaged secondary‐level correlations. Physical and financial inputs were the highest‐ranking primary input categories, while nursing quality/safety and service outputs ranked highest among primary output categories (Table 3). At the secondary level, strongest‐correlated measurable inputs were actual open beds, nurses on duty, costs of nursing staff, and nurse–patient ratios; strongest outputs were nursing quality assessment results, bed utilization rate, patient satisfaction, case‐mix index, and number of diagnosis‐related groups.

**TABLE 3:** Gray relation analysis of time series results.

**Primary input influence factors**	**Gray correlation coefficient**	**Rank**	**Secondary input influence factors**	**Gray correlation coefficient**	**Rank**

Manpower inputs	0.9237	3	Number of nurses on duty	0.9302	2
			Number of day shift nurses	0.9083	10
			Number of night shift nurses	0.9231	8
			Doctor–nurse ratio	0.9244	6
			Nurse–patient ratio	0.9281	4
			Bed‐to‐nurse ratio	0.9281	5

Physical inputs	1.0000	1	Actual number of open beds	1.0000	1

Financial inputs	0.9299	2	Costs of nursing staff	0.9299	3

Nursing service inputs	0.8959	4	Nursing work hours	0.9122	9
			Nursing work hours at night	0.9231	7
			Nursing hours per patient per day	0.8526	11

**Primary output influence factors**	**Gray correlation coefficient**	**Rank**	**Secondary output influence factors**	**Gray correlation coefficient**	**Rank**

Nursing services outputs	0.8514	1	Number of diagnosis‐related groups	0.8983	5
			Case‐mix index	0.9027	4
			Number of relative weights ≥ 2	0.7881	12
			Actual number of occupied bed days	0.8680	6
			Average length of stay for discharged patients	0.8192	11
			Number of bed turnovers	0.8240	9
			Bed utilization rate	0.9803	2
			Number of discharged patients	0.8242	8
			Discharge rate of critical patients	0.8563	7
			Number of surgeries	0.8192	10
			Surgical outputs	0.7853	13

Nursing safety and quality outputs	0.8381	2	Adverse nursing events	0.5852	15
			Nursing quality assessment results	0.9909	1
			Patient satisfaction	0.9383	3

Scientific research outputs	0.7163	3	Scientific research achievements	0.7163	14

### 3.3. Results of Gray Relation Analysis in Nursing Unit Series

The nursing unit series analysis showed analogous differentiation, with primary indicators exhibited higher composite correlation coefficients (0.8329–1.0000) than secondary indicators (0.7971–1.0000), reflecting their calculation as averages of secondary indicator relationships. Physical/financial inputs and combined quality/safety‐service outputs were again the highest‐ranking primary categories (Table 4). Among secondary variables, actual open beds, number of nurses on duty, nurse–patient ratio, and nursing work hours showed strongest input correlations, while nursing quality assessment results, bed utilization rate, patient satisfaction, actual number of occupied bed days, and diagnosis‐related groups demonstrated strongest output correlations. The most consistently correlated secondary inputs across both analytical dimensions were actual open beds, nurses on duty, nurse–patient ratio, and nursing work hours; strongest secondary outputs were nursing quality assessment results, bed utilization rate, patient satisfaction, and diagnosis‐related groups. Adverse nursing events exhibited divergent secondary‐level correlations across analytical dimensions, demonstrating higher coefficients in nursing unit comparisons (*γ* = 0.88) than in the time series analysis (*γ* = 0.62).

**TABLE 4:** Gray relation analysis of nursing units series results.

**Primary input influence factors**	**Gray correlation coefficient**	**Rank**	**Secondary input influence factors**	**Gray correlation coefficient**	**Rank**

Manpower inputs	0.9477	3	Number of nurses on duty	0.9635	2
			Number of day shift nurses	0.9600	7
			Number of night shift nurses	0.9095	10
			Doctor–nurse ratio	0.9261	9
			Nurse–patient ratio	0.9635	2
			Bed‐to‐nurse ratio	0.9635	2

Physical inputs	1.0000	1	Actual number of open beds	1.0000	1

Financial inputs	0.9573	2	Costs of nursing staff	0.9574	8

Nursing service inputs	0.9449	4	Nursing work hours	0.9635	5
			Nursing work hours at night	0.9095	11
			Nursing hours per patient per day	0.9616	6

**Primary output influence factors**	**Gray correlation coefficient**	**Rank**	**Secondary output influence factors**	**Gray correlation coefficient**	**Rank**

Nursing services outputs	0.8801	2	Number of diagnosis‐related groups	0.9279	5
			Case‐mix index	0.8958	9
			Number of relative weights ≥ 2	0.7818	14
			Actual number of occupied bed days	0.9688	4
			Average length of stay for discharged patients	0.8957	10
			Number of bed turnovers	0.9050	7
			Bed utilization rate	0.9832	1
			Number of discharged patients	0.9022	8
			Discharge rate of critical patients	0.7750	15
			Number of surgeries	0.8492	11
			Surgical outputs	0.7971	13

Nursing safety and quality outputs	0.9615	1	Adverse nursing events	0.9183	6
			Nursing quality assessment results	0.9832	1
			Patient satisfaction	0.9830	3

Scientific research outputs	0.8329	3	Scientific research achievements	0.8329	12

## 4. Discussion

This GRA study quantified the relative strength of association between diverse factors and nurse staffing across temporal and structural dimensions. The core findings reveal distinct hierarchical patterns and identify key drivers, providing novel insights for nurse staffing theory and actionable guidance for management practice. Crucially, primary indicators (aggregate measures) consistently demonstrated stronger correlations with staffing than secondary indicators (directly measured variables) in both time series and nursing unit analyses. This empirically validates their conceptualization as stable, systemic representations of underlying constructs, making them particularly valuable for high‐level strategic decision‐making regarding resource allocation and outcome expectations. This pattern of findings strongly aligns with the Donabedian SPO framework that guided this study. Within the SPO model, these primary indicators effectively operationalize key “Structure” elements (inputs/resources) and “Outcome” domains, while their robust association with nursing staffing underscores its central role as a vital “Process” that links structural inputs to care outcomes.

The identification of physical/financial inputs as the dominant primary input category across both analytical dimensions underscores their fundamental role as boundary conditions shaping staffing possibilities. These inputs, particularly the actual open beds which emerged as the most consistent and strongly correlated secondary input, represent the tangible capacity constraints within which staffing must operate [34]. In Donabedian terms, these are core structural elements that set the stage for care. Concurrently, combined nursing quality/safety and service outputs consistently ranked highest among primary output categories, especially in the nursing unit analysis. This highlights that achieving optimal outcomes in patient safety, service efficiency (e.g., bed utilization rate), and managing case complexity (via diagnosis‐related groups/case‐mix index) is most sensitive to appropriate staffing levels, emphasizing the intrinsic link between staffing adequacy and core nursing objectives [35–37]. The strong correlations observed between these structural inputs and outcome categories provide empirical support for the proposed linkages within the SPO model, where structure enables outcomes.

Delving into the secondary indicators clarifies the specific, actionable metrics with the strongest influence. Alongside actual open beds, number of nurses on duty, nurse–patient ratios and nursing work hours were consistently highly correlated inputs, reinforcing their direct relevance to meeting patient care demands structurally and volumetrically [34–36]. These indicators pertain to the structural capacity for process. Among outputs, nursing quality assessment maintained the strongest correlation, directly linking staffing levels to care quality perceptions and measurements. The prominence of diagnosis‐related groups/case‐mix index provides critical quantitative evidence that staffing must account for the complexity and diversity of patient conditions to ensure effective, efficient, and economically viable care delivery [38–40]. These findings underscore that staffing, as a structural factor, is profoundly connected to outcome measures of quality, efficiency, and case complexity. The significant divergence in correlation strength for adverse nursing events between the nursing unit analysis and the time series analysis is a pivotal finding. This indicates that staffing adequacy has a far more direct and substantial impact on safety outcomes within specific unit contexts than suggested by aggregated temporal trends, likely because time‐series averages mask significant interunit variability in both adverse events and staffing adequacy. This dimensional variation enriches the SPO model by suggesting that the strength of the structure–outcome relationship may be context‐dependent and most salient when analyzed at the appropriate operational unit level, rather than through aggregated temporal data which may obscure critical unit‐level dynamics.

These findings translate into concrete imperatives for nurse managers. First, decision‐making must explicitly acknowledge the paramount influence of physical/financial input constraints (actual open beds, staffing budgets) as the foundational boundaries [34, 41]. Staffing plans must operate realistically within these limits, avoiding both wasteful overexpansion without quality outcomes and demoralizing underinvestment that fuels burnout and turnover [42–45]. Second, staffing strategies should be fundamentally outcome‐focused, prioritizing achieving targets in combined quality/safety and service outputs. Managers must explicitly link staffing levels to mitigating adverse events (monitored rigorously at the unit level), sustaining quality ratings, effectively managing patient complexity (diagnosis‐related groups), and optimizing resource utilization (bed utilization rate). Third, the correlation strengths provide a clear hierarchy for metric prioritization. Actual open beds, nurse–patient ratios, and nursing work hours should be core monitored input indicators, while nursing quality assessment and diagnosis‐related groups/case‐mix index should be central output indicators for staffing dashboards and decision‐support systems. This aligns with a value‐driven interpretation of the SPO model, where structural investments are evaluated based on the outcomes they produce.

Furthermore, the robust link between staffing levels, case complexity, and quality/safety outputs provides compelling evidence to shift the managerial paradigm from cost cutting to value creation. Adequate staffing calibrated to patient acuity and diversity is an investment that enhances efficiency (preventing costly errors and reducing length of stay), improves outcomes, and supports revenue generation through effective complex case management [4, 35, 46]. This value‐generation perspective counters the short‐sighted view of staffing as a pure cost center and argues for sufficient investment to achieve sustainable quality and economic efficiency [47]. Importantly, the stark contrast in adverse event correlations necessitates unit‐specific staffing analysis and planning. Aggregated organizational data are insufficient for managing the safety risks intrinsically tied to staffing adequacy within distinct unit environments. This supports a nuanced application of the SPO framework, where the model’s relationships are validated and must be managed at the basic system level.

The GRA findings significantly simplify the complex landscape of influencing factors by identifying high‐priority drivers. Future research should leverage this clarity to develop predictive staffing models using these key indicators (actual open beds, number of nurses on duty, work hours, quality assessment, and diagnosis‐related groups) as foundational inputs. Building integrated information technology systems to maintain real‐time databases of these prioritized metrics is essential [48]. Crucially, simulation models informed by the relative priority and strength of association established in this study can enable the construction of differentiated, evidence‐based staffing plans optimized for the unique demands of various nursing unit types (e.g., high‐acuity intensive care units vs. general wards), moving decisively beyond rigid, one‐size‐fits‐all approaches.

### 4.1. Limitation

There are some limitations of this study. As this is a single‐center study with a relatively small sample size, the sample size and the scope of the study could be expanded in the future to explore the different correlations of factors affecting the staffing of nursing units between different regions or countries. Moreover, this study only explores the correlation between the main factors and nursing unit staffing, after which a nursing unit human resource efficiency evaluation system can be constructed based on the influential factors with strong correlation. At the same time, an information system can be established to provide decision support for managers to construct a practical nursing staffing model.

## 5. Conclusion

This GRA quantified the relative influence of factors on nursing staffing, revealing that aggregate primary indicators exhibited stronger associations than secondary variables, affirming their value for strategic decisions. Physical/financial inputs, particularly the fundamental constraint of actual open beds, constituted the paramount input category, defining operational boundaries. Conversely, combined nursing quality/safety and service outputs emerged as the most influential outcome category, demonstrating their heightened sensitivity to staffing adequacy. Critically, actual open beds, number of nurses on duty, and nursing work hours were the strongest measurable input drivers, while nursing quality assessment, bed utilization rate, and diagnosis‐related groups ranked highest among outputs, underscoring the necessity to calibrate staffing to patient complexity for quality and efficiency. These findings, interpreted through the lens of the Donabedian SPO framework, confirm staffing as a pivotal “Process” that is fundamentally shaped by core “Structure” elements (e.g., physical/financial inputs) and is critically linked to achieving desired “Outcomes” in quality, safety, and service efficiency. Effective staffing therefore necessitates a value‐driven approach operating within physical/financial constraints while prioritizing investments linked directly to achieving key quality, safety, and service efficiency outcomes. Managers must prioritize these high‐impact indicators for monitoring and modeling, tailoring plans to unit‐specific contexts as evidenced by the pronounced unit‐level impact on adverse events. Future efforts should leverage these prioritized factors to develop predictive models and simulation tools for constructing optimized, evidence‐based staffing plans across diverse nursing units, enhancing both clinical effectiveness and economic sustainability.

## Funding

This study was funded by the Health Commission of Zhejiang Province (grant number: 2024KY146) and Zhejiang Kang Enbei Hospital Management Soft Science Research Project (grant number: 2023ZHA‐KEB102).

## Conflicts of Interest

The authors declare no conflicts of interest.

## Data Availability

The data that support the findings of this study are available on request from the corresponding author. The data are not publicly available due to privacy or ethical restrictions.
